# Development and validation of an interpretable machine learning model for predicting chemotherapy-induced neutropenia in small cell lung cancer: a web-based clinical decision support tool

**DOI:** 10.3389/fonc.2026.1939436

**Published:** 2026-09-04

**Authors:** Jingyue Zhang, Siyu Lu, Chang Liu, Yang Zhai, Jiahui Liu, Miaomiao Luo, Hanxu Zhang, Shijiao Cai, Ye Tian, Linlin Zhang, Hengjie Yuan

**Affiliations:** 1 Department of Pharmacy, Tianjin Medical University General Hospital, Tianjin, China; 2 Department of Medical Oncology, Tianjin Medical University General Hospital, Tianjin, China; 3 Department of Neurosurgery, Tianjin Medical University General Hospital, Tianjin, China

**Keywords:** chemotherapy-induced neutropenia, clinical decision support system, machine learning, predictive modeling, small cell lung cancer

## Abstract

**Objective:**

To develop and validate an interpretable machine learning model for predicting grade ≥3 chemotherapy-induced neutropenia (CIN) in patients with small cell lung cancer (SCLC), and to establish a web-based clinical decision support tool for individualized risk assessment.

**Methods:**

We conducted a retrospective observational analysis of prospectively collected clinical data and enrolled 209 patients with SCLC who underwent 840 chemotherapy cycles between 2019 and 2022 from Tianjin Medical University General Hospital. Patients rather than chemotherapy cycles were randomly assigned into the training (80%) and testing (20%) cohorts. Feature selection was performed using least absolute shrinkage and selection operator regression and the Boruta algorithm. Machine learning algorithms were compared using patient-level 5-fold GroupKFold cross-validation. Hyperparameters were optimized using Optuna, and model interpretability was evaluated using Shapley additive explanations, Local interpretable model-agnostic explanations, Partial dependence plots. Three clinically oriented probability thresholds were further assessed to support different clinical decision-making scenarios. A web-based clinical decision support tool was subsequently developed.

**Results:**

CatBoost achieved the highest cross-validation performance while demonstrating the most stable results across folds, and was selected as the final model. Five predictors were selected for model development, including chemotherapy cycle, chemotherapy regimen, prophylactic medication, absolute neutrophil count (ANC) and rescue treatment in the prior cycle. In the independent testing cohort, the optimized CatBoost model achieved an AUC of 0.785 (95% CI, 0.706–0.865), with good calibration and favorable clinical net benefit. Threshold-specific analyses illustrated the expected sensitivity–specificity trade-offs for exploratory screening- and confirmatory-oriented operating points. Interpretability analyses identified early chemotherapy cycles, rescue treatment in the prior cycle, absence of prophylactic medication, VP16 plus platinum regimen, and lower baseline ANC as predictors associated with higher CIN risk in this dataset. The model was further deployed as an interactive web-based application, available at http://39.96.172.15:8080/.

**Conclusions:**

An interpretable CatBoost model was developed for predicting grade ≥3 CIN in patients with SCLC. The integration of the model with a web-based clinical decision support tool may facilitate individualized risk stratification.

## Introduction

Small cell lung cancer (SCLC) accounts for approximately 15% of all lung cancers and is characterized by rapid progression, early metastasis, and poor prognosis (1). Despite recent therapeutic advances, systemic chemotherapy remains the cornerstone of treatment for both limited- and extensive-stage disease (2). However, chemotherapy-induced neutropenia (CIN) is one of the most common dose-limiting toxicities and frequently results in treatment delays, dose reductions, infectious complications, and even treatment-related mortality (3, 4). Although prophylactic granulocyte colony-stimulating factor (G-CSF) is recommended for patients at high risk of CIN, identifying those most likely to benefit from preventive intervention remains challenging in routine clinical practice (5).

Several prediction models have been developed to estimate CIN risk. Conventional logistic regression models and clinical risk scores are generally limited by linear assumptions and inability to capture complex interactions among clinical variables (6–8). Machine learning approaches have shown improved predictive performance by modeling nonlinear relationships (9, 10). However, most published models were developed in heterogeneous cancer populations, lacked disease-specific focus on SCLC, or provided limited model interpretability (9, 10). Furthermore, few studies have focused specifically on clinically significant grade ≥3 neutropenia, and even fewer have translated prediction models into practical clinical decision support tools that facilitate real-time risk assessment.

Therefore, this study aimed to develop and validate an interpretable machine learning model for predicting grade ≥3 CIN in patients with SCLC. Model interpretability was further assessed using Shapley additive explanations (SHAP), Local interpretable model-agnostic explanations (LIME), and Partial dependence plots (PDP) to characterize feature contributions and enhance transparency. We also developed a user-friendly web-based clinical decision support tool to enable individualized, real-time CIN risk assessment and facilitate risk stratification.

## Methods

### Study population and data collection

This study was a retrospective analysis of prospectively collected routine clinical data conducted at the Department of Medical Oncology, Tianjin Medical University General Hospital, China. Consecutive patients with histologically or cytologically confirmed SCLC who received systemic chemotherapy between January 2019 and December 2022 were enrolled. The exclusion criteria were as follows (1): age <18 years (2); absence of a confirmed malignancy (3); pregnancy or lactation (4); concurrent radiotherapy (5); prior history of stem cell transplantation (6); diagnosis of acute leukemia (7); presence of grade ≥2 neutropenia before initiation of chemotherapy; or (8) patients with a combination of other tumors. This study consisted of a retrospective analysis of prospectively collected clinical data, and ethical approval was granted with waiver of informed consent due to its retrospective observational nature.

Data were prospectively documented in the study-specific research database during routine clinical practice, including patient demographics, comorbidities, treatment information, and baseline laboratory parameters prior to chemotherapy. Demographic and clinical variables included gender, age, anemia, hypertension, lung disease, diabetes, thyroid disease, heart failure, rheumatoid arthritis, kidney disease, liver disease, autoimmune disease, coronary heart disease, and bone metastasis. Treatment-related variables included body surface area, chemotherapy regimen, chemotherapy cycle, prophylactic medication, CIN in the prior cycle, rescue treatment in the prior cycle, and chemotherapy repetition cycle. Baseline laboratory measurements obtained before chemotherapy included aspartate aminotransferase, alanine aminotransferase, alkaline phosphatase, gamma-glutamyl transferase, plasma bilirubin, total protein, albumin, serum creatinine, glomerular filtration rate, leukocyte count, absolute neutrophil count (ANC), lymphocyte count, blood hemoglobin, and thrombocyte count.

The primary outcome was the occurrence of grade ≥3 CIN, defined according to the Common Terminology Criteria for Adverse Events (CTCAE, version 5.0) (11). Neutropenia was assessed based on routine blood examinations during each chemotherapy cycle.

### Data splitting and preprocessing

Patients rather than chemotherapy cycles were randomly assigned into the training (80%) and testing (20%) cohorts to avoid patient-level information leakage. Missing data were handled using a structured approach. Variables with more than 20% missing values were excluded from further analysis to reduce potential bias associated with imputation. For the remaining variables, missing values were imputed using Multivariate Imputation by Chained Equations (MICE) (12). To prevent information leakage during model development, the imputation model was fitted independently within each training fold during cross-validation and subsequently applied to the corresponding validation fold using the parameters learned exclusively from that training fold. After model selection and hyperparameter optimization (HPO), the imputation model was refitted using the full training cohort and then applied once to the independent testing cohort. Detailed information regarding the extent of missingness for each variable was provided in Supplementary Table I. Outliers were identified using the Isolation Forest algorithm. During cross-validation, the Isolation Forest model was independently fitted using only the observations in each training fold. Samples identified as outliers were removed only from the corresponding training fold, whereas the validation fold was retained unchanged to ensure that validation performance was evaluated on unseen observations without data-dependent exclusion. After model selection and HPO, the Isolation Forest model was refitted using the full training cohort, and the independent testing cohort was left unchanged to preserve its original distribution and avoid optimistic performance estimation.

To identify robust predictors of CIN, a two-step feature selection strategy was employed. First, least absolute shrinkage and selection operator (LASSO) (13) regression was applied to perform variable selection and shrinkage, reducing model complexity by penalizing less informative features. Second, the Boruta algorithm (14), a wrapper-based feature selection method built upon random forests, was used to identify all relevant features by comparing the importance of original variables with that of randomly permuted shadow features. During cross-validation, both LASSO and Boruta were independently refitted using only the data available in each training fold. The features selected by both methods were retained for that fold and subsequently applied unchanged to the corresponding validation fold. Thus, no information from a validation fold was used for feature selection, and validation samples remained completely unseen during these data-dependent procedures. Following model selection and HPO, the feature-selection procedures were refitted using the full training cohort, and the resulting final predictor set was subsequently applied to the independent testing cohort.

Categorical variables were handled according to their measurement level. Nominal categorical variables, including chemotherapy regimen, were explicitly treated as categorical predictors in CatBoost rather than as continuous numerical variables. For algorithms requiring numerical input, categorical variables were appropriately encoded using transformations fitted exclusively within the corresponding training folds and subsequently applied to the validation folds. Supplementary Table II presents the digital conversion of categorical variables.

### Model development and evaluation

To compare the predictive performance of different machine learning algorithms, ten classification models were developed. The algorithms included extreme gradient boosting (XGBoost), light gradient boosting machine (LightGBM), adaptive boosting (AdaBoost), multilayer perceptron (MLP), k-nearest neighbors (KNN), Naïve Bayes, decision tree, random forest, support vector machine (SVM), and categorical boosting (CatBoost) (15, 16). Model development was performed exclusively within the training cohort using 5-fold patient-level GroupKFold cross-validation. Patient identifiers were used as grouping variables to ensure that all chemotherapy cycles from the same patient were assigned to the same fold, thereby preventing information leakage between training and validation subsets. For algorithms sensitive to feature scaling (KNN, SVM, and MLP), feature standardization was performed separately within each training fold using a StandardScaler fitted only on the training data, and the fitted scaler was subsequently applied to the corresponding validation fold. Tree-based algorithms (XGBoost, LightGBM, CatBoost, random forest, AdaBoost, and decision tree) and Gaussian Naïve Bayes were trained using the original feature values without normalization. The machine learning algorithm demonstrating the highest mean area under the receiver operating characteristic curve (AUC) together with stable performance across cross-validation folds was selected as the candidate model for subsequent HPO and independent testing. HPO was performed using the Optuna framework (version 4.9.0) (17) with 200 optimization trials. The optimal hyperparameters for the final model were selected according to mean AUC across the five validation folds. After model selection and HPO were completed, the finalized preprocessing, feature-selection, and modeling pipeline was refitted using the entire training cohort.

Three exploratory operating probability thresholds were evaluated to illustrate how the model could be used under different clinical objectives. The first threshold was determined using the Youden index to maximize the combined sensitivity and specificity. The second threshold was selected using a pragmatic screening-oriented operating target of sensitivity ≥0.75, with specificity maximized among thresholds meeting this criterion. The third threshold was selected using a pragmatic confirmatory-oriented operating target of specificity ≥0.80, with sensitivity maximized among thresholds meeting this criterion. These operating targets were chosen to represent relatively high sensitivity for screening and relatively high specificity for confirmatory assessment, respectively, and were not intended to represent clinically established decision thresholds. All thresholds were determined from the training cohort and were subsequently applied unchanged to the independent testing cohort, where performance was reported as observed.

Model performance was evaluated in the testing cohort. Discrimination was assessed using the AUC with 95% confidence intervals (CI). CIs were estimated by patient-level cluster bootstrap resampling (2000 iterations), in which patients—rather than individual chemotherapy cycles—were resampled to account for within-patient correlation. Additionally, a confounding matrix was constructed to quantify classification performance, including true positives (TP), true negatives (TN), false positives (FP), and false negatives (FN). Based on these components, evaluation metrics—including accuracy, sensitivity, specificity, precision, and F1 score—were calculated to provide a comprehensive assessment of model performance. Detailed formulas for these metrics were provided in Supplementary Table III. Calibration was assessed using calibration curves and the Brier score. Decision curve analysis (DCA) was conducted to evaluate the clinical utility of the model across a range of threshold probabilities (18).

**Figure 1:**
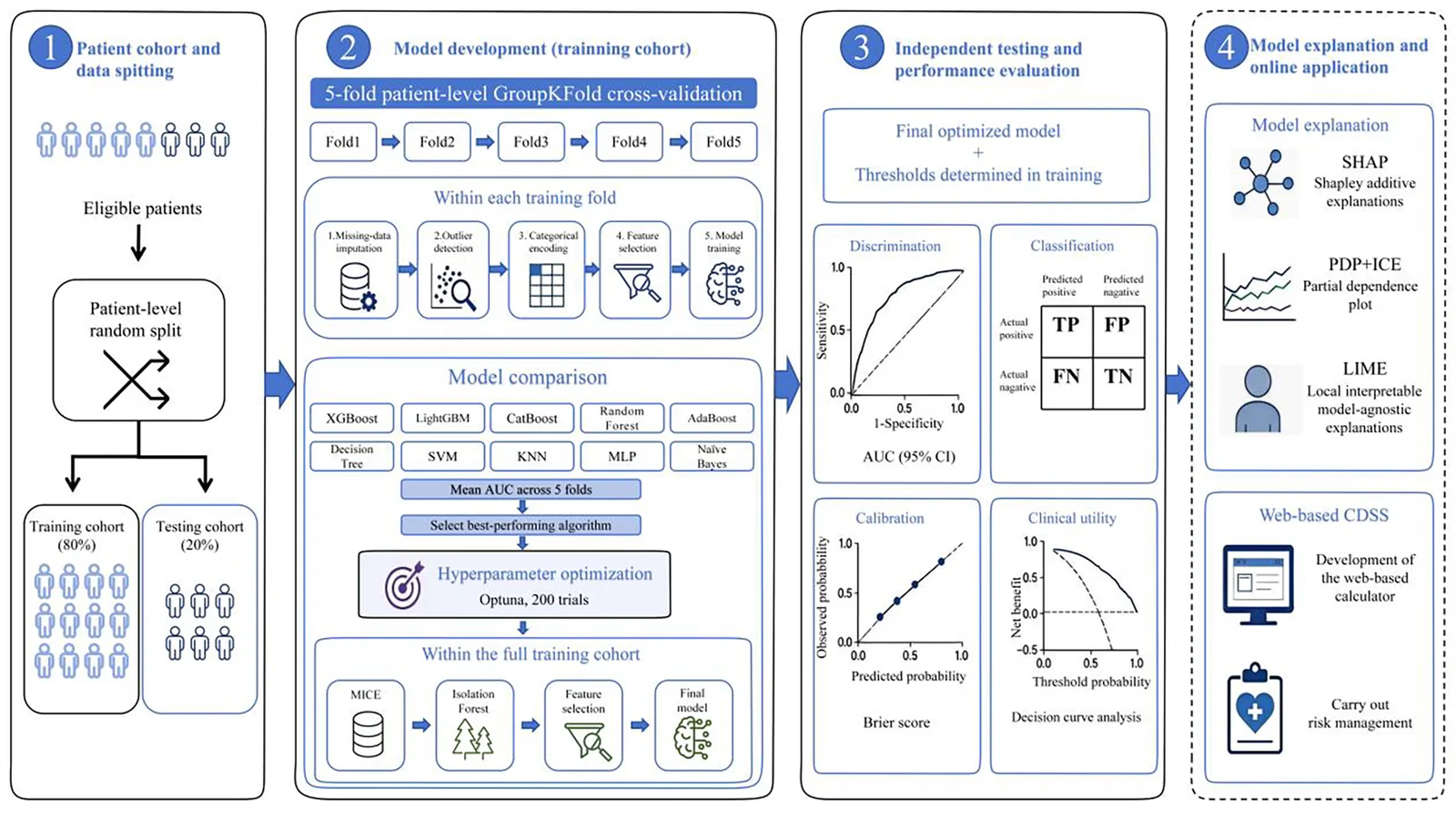
Research process flow diagram.

To avoid the contingency caused by a single interpretation technique, several explainable machine learning techniques were applied. SHAP (19) were used to quantify feature importance and visualize both global and individual-level contributions. LIME (20) were used to provide case-specific explanations. PDP (21) were generated to illustrate the marginal effects of key predictors. The research process is illustrated in **Figure 1**.

### Web-based clinical decision support tool

The online application was developed using a Vue-based frontend and a Python-based backend. The system comprises three functional modules designed to support individualized risk assessment, model interpretation, and risk stratification. A core component of the platform is an interactive input interface that allows users to select and modify five clinically relevant variables, enabling patient-level configuration. Based on user-defined inputs, a dedicated prediction module estimates the probability of grade ≥3 CIN, providing quantitative risk outputs. To improve model interpretability, a dedicated explanation module was integrated using SHAP. This module quantitatively illustrates the contribution of each predictor to the final prediction at individual levels, displaying the contribution and direction of each input feature to the model’s prediction for that patient. In addition, the interface displays categories based on a prespecified training-derived exploratory operating threshold.

### Statistical analysis

All statistical analyses and machine learning procedures, including model development and hyperparameter tuning, were performed using Python (version 3.10.20; Python Software Foundation) with the scikit-learn library. Continuous variables are presented as mean ± standard deviation (SD) or median with interquartile range (IQR), as appropriate based on their distribution. Categorical variables are summarized as frequencies and percentages.

## Results

### Baseline characteristics

A total of 209 patients contributing 840 chemotherapy cycles were included in the study. Patients were randomly assigned at the patient level to the training cohort (80%) and testing cohort (20%), with all chemotherapy cycles from the same patient retained within the same cohort. The training cohort comprised 167 patients and 662 chemotherapy cycles, of which 179 cycles (27.04%) were complicated by grade ≥3 CIN. The testing cohort comprised 42 patients and 178 chemotherapy cycles, including 52 cycles (29.21%) with grade ≥3 CIN. Among these patients, 93 (55.69%) patients in the training cohort and 25 (59.52%) patients in the testing cohort experienced at least one grade ≥3 CIN event. The median number of chemotherapy cycles per patient was 4 (range 1-13) in the training cohort and 4 (range 1-9) in the testing cohort.

Overall, grade ≥3 CIN occurred in 231 of 840 chemotherapy cycles (27.50%). The median age of the patients was 65 years, and 79.43% were male. Approximately 43.06% of patients had hypertension, 20.57% had diabetes, and 28.71% had bone metastasis. The most frequently administered chemotherapy regimens were etoposide (VP16) plus cisplatin (DDP) and etoposide (VP16) plus carboplatin (CBP), accounting for 37.02% and 25.60% of chemotherapy cycles, respectively. Details of the clinical characteristics are shown in **Table 1**.

**Table 1:** Patient and treatment characteristics.

Patient characteristic	Patients (n=209)	Training cohort (n=167)	Testing cohort (n=42)
Age (years), median (IQR)	65 (58–70)	65 (58–70)	65 (59–69)
Male, n (%)	166(79.43%)	133(79.64%)	33(78.57%)
Experienced at least one grade ≥3 CIN event, n (%)	118(56.46%)	93(55.69%)	25(59.52%)
Number of chemotherapy cycles, n (%)	4 (1–13)	4 (1–13)	4 (1–9)
Comorbidities, n (%)			
Anemia	14(6.70%)	13(7.78%)	1(2.38%)
Hypertension	90(43.06%)	73(43.71%)	17(40.48%)
Lung disease	72(34.45%)	59(35.33%)	13(30.95%)
Diabetes	43(20.57%)	35(20.96%)	8(19.05%)
Thyroid disease	16(7.66%)	12(7.19%)	4(9.52%)
Heart failure	3(1.44%)	1(0.60%)	2(4.76%)
Rheumatoid arthritis	1(0.48%)	1(0.60%)	0(0.00%)
Kidney disease	21(10.05%)	17(10.18%)	4(9.52%)
Liver disease	45(21.53%)	37(22.16%)	8(19.05%)
Autoimmune disease	1(0.48%)	1(0.60%)	0(0.00%)
Coronary heart disease	37(17.70%)	30(17.96%)	7(16.67%)
Bone metastasis	60(28.71%)	52(31.14%)	8(19.05%)
Patient/treatment characteristic	All cycles (n=840)	Training cohort (n=662)	Testing cohort (n=178)
Body surface area, mean ± (SD)	1.82 ± 0.17	1.82 ± 0.18	1.83 ± 0.13
Chemotherapy cycles, median (range)	3 (1–13)	3 (1–13)	3 (1–9)
Prophylactic medication, n (%)	211(25.12%)	174(26.28%)	37(20.79%)
CIN in the prior cycle, n (%)	360(42.86%)	273(41.24%)	87(48.88%)
Rescue treatment in the prior cycle, n (%)	212(25.24%)	161(24.32%)	51(28.65%)
Chemotherapy regimen, n (%)			
CPT-11 based	101(12.02%)	83(12.54%)	18(10.11%)
CPT-11 plus CBP based	13(1.55%)	13(1.96%)	0(0.00%)
CPT-11 plus DDP based	11(1.31%)	11(1.66%)	0(0.00%)
CPT-11 plus LBP based	2(0.24%)	2(0.30%)	0(0.00%)
VP16 based	27(3.21%)	19(2.87%)	8(4.49%)
VP16 plus CBP based	215(25.60%)	174(26.28%)	41(23.03%)
VP16 plus DDP based	311(37.02%)	257(38.82%)	54(30.34%)
VP16 plus LBP based	120(14.29%)	76(11.48%)	44(24.72%)
VP16 plus NDP based	3(0.36%)	2(0.30%)	1(0.56%)
Doc based	32(3.81%)	24(3.63%)	8(4.49%)
PTX based	5(0.60%)	1(0.15%)	4(2.25%)
Aspartate aminotransferase, median (IQR)	20.00 (16.00-26.00)	20.00(16.00-26.00)	20.00(16.00-26.00)
Alanine aminotransferase, median (IQR)	17.00 (13.00-28.00)	17.00(13.00-27.00)	19.00(13.00-31.50)
Alkaline phosphatase, median (IQR)	86.00 (70.00-107.00)	85.00(70.25-106.75)	91.00(70.50-107.50)
Gamma-glutamyl transferase, median (IQR)	42.00 (28.00-70.00)	42.00(28.00-69.00)	43.00(27.50-77.00)
Plasma bilirubin, median (IQR)	8.70 (6.60-11.30)	8.55(6.60-11.40)	8.90(6.65-11.05)
Total Protein, mean ± (SD)	69.06 ± 6.32	69.30 ± 6.21	68.21 ± 6.67
Albumin, median (IQR)	38.00 (35.00-41.00)	38.00(35.00-41.00)	39.00(35.00-42.00)
Serum creatinine (IQR)	62.00 (50.00-75.00)	62.00(50.00-76.00)	59.50(50.00-69.00)
Glomerular filtration rate, median (IQR)	98.25 (89.07-105.87)	97.46(87.11-102.25)	92.75(89.31-96.18)
Leukocyte count, median (IQR)	6.18 (4.80-7.88)	6.20(4.86-7.85)	6.04(4.55-8.24)
Absolute neutrophil count, median (IQR)	4.06 (2.94-5.59)	4.12(2.99-5.55)	3.85(2.81-5.85)
Lymphocyte count, median (IQR)	1.24 (0.93-1.68)	1.25(0.94-1.69)	1.17(0.90-1.65)
Blood hemoglobin, mean ± (SD)	119.93 ± 17.26	119.23 ± 17.49	122.53 ± 16.22
Thrombocyte count, median (IQR)	215.50 (166.00-276.25)	221.00(164.75-280.00)	196.00(168.00-259.75)
grade ≥3 CIN, n (%)	231(27.50%)	179(27.04%)	52(29.21%)

CBP, carboplatin; CPT-11, irinotecan; DDP, cisplatin; Doc, docetaxel; IQR, interquartile range; LBP, lobaplatin; NDP, nedaplatin; PTX, paclitaxel; VP16, etoposide; SD, standard deviation.

### Model selection

**Figure 2:**
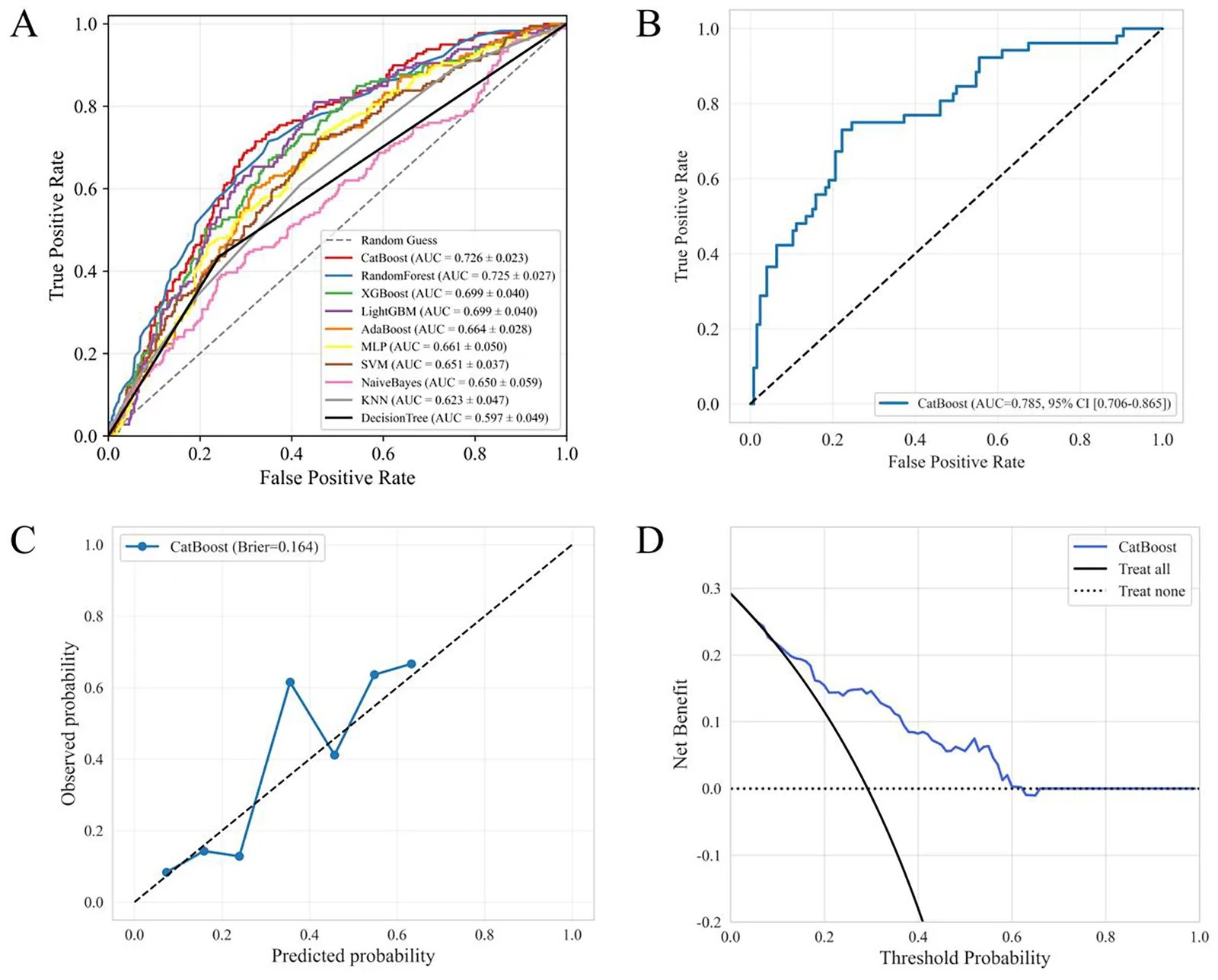
Performance of the models. **(A)** Performance comparison of the 10 models using patient-level 5-fold GroupKFold cross-validation., **(B)** ROC curves of the optimized CatBoost model in testing cohort; **(C)** calibration curves of the optimized CatBoost model in testing cohort, **(D)** DCA curve of the optimized CatBoost model in testing cohort. AdaBoost, adaptive boosting; AUC, area under the receiver operating characteristic curve; CatBoost, categorical boosting; DCA, Decision curve analysis; XGBoost, extreme gradient boosting; KNN, k-nearest neighbors; LightGBM, light gradient boosting machine; MLP, multilayer perceptron; ROC, receiver operating characteristic; SVM, and support vector machine.

Ten machine learning algorithms were evaluated using 5-fold patient-level GroupKFold cross-validation. Among the evaluated models, CatBoost achieved the highest mean AUC while demonstrating the most consistent discrimination across validation folds, indicating good predictive performance and robustness (**Figure 2A**). Therefore, CatBoost was selected as the optimal algorithm for subsequent hyperparameter optimization and independent evaluation on the held-out testing cohort.

### Model construction and performance

**Figure 3:**
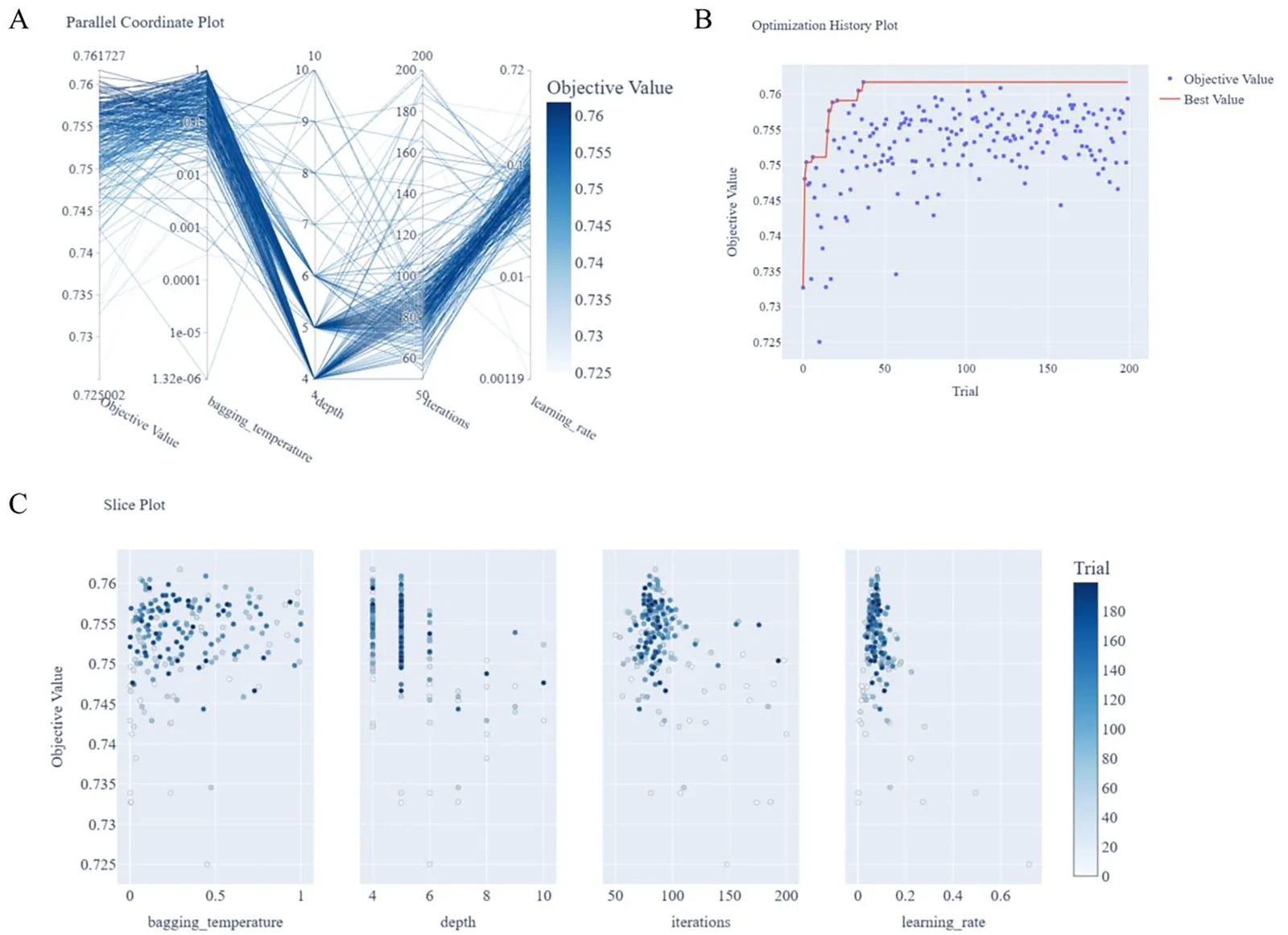
HPO process for the CatBoost model. **(A)** Parallel coordinate plot showing the distribution of hyperparameters in the CatBoost model during HPO, where dark colors are matched with higher AUC values; **(B)** optimization history plot showing the evolution of the best objective values with the number of trials; **(C)** slice plot showing the correlation between each hyperparameter and AUC. AUC, area under the receiver operating characteristic curve; HPO, hyperparameter optimization.

HPO was performed on the CatBoost model, and the hyperparameter combinations with the highest mean AUC score in cross-validation were identified after 200 trials. **Figure 3** illustrates the HPO process for the CatBoost model. The CatBoost model was finally trained with the following hyperparameter settings: iterations: 85, learning_rate: 0.08297564383082118, depth: 4, bagging_temperature: 0.2924462884260101.

**Figure 4:**
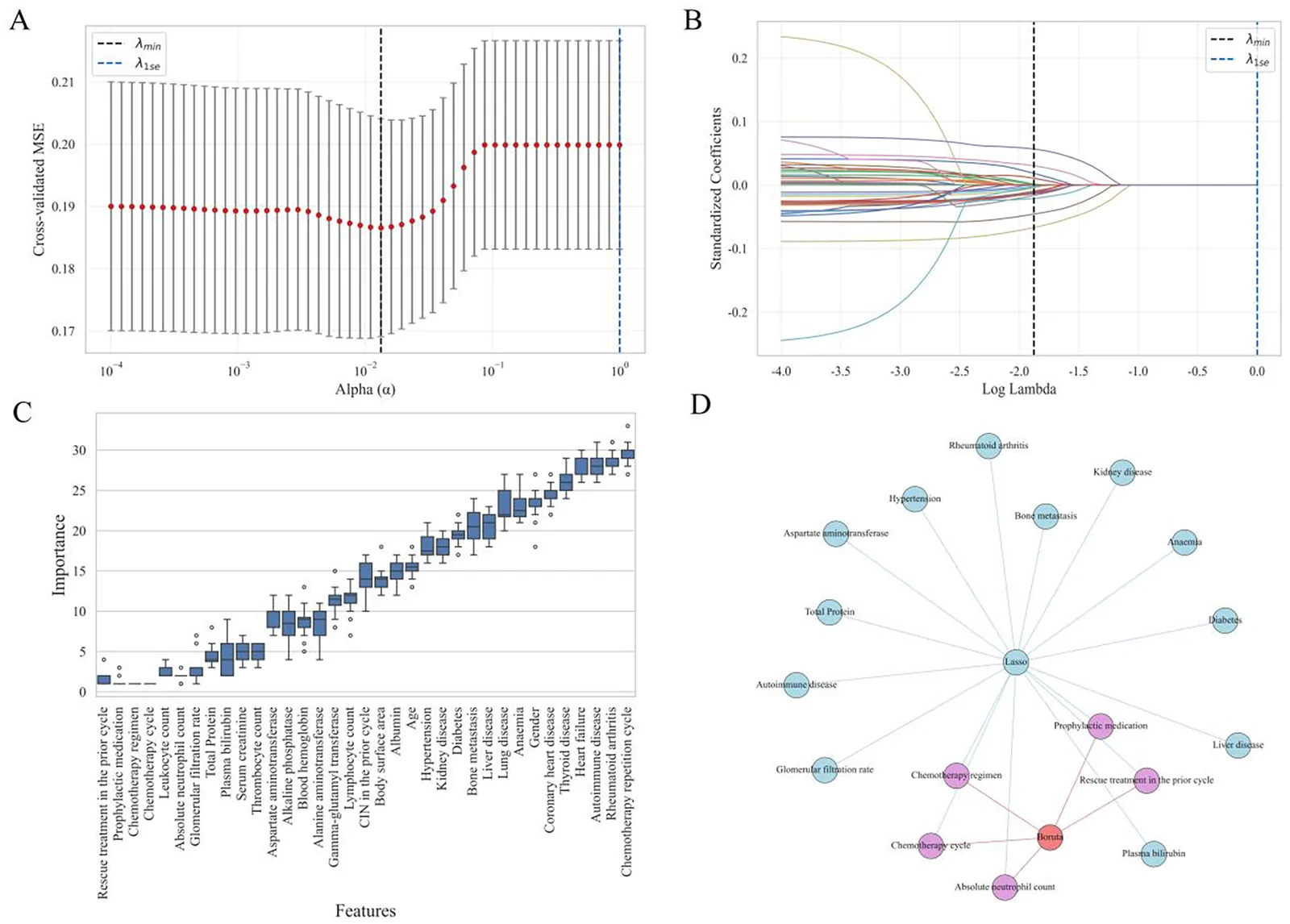
Predictor screening results. **(A)** Factor screening based on the LASSO regression model, with the left dashed line indicating the best lambda value for the evaluation metrics (lambda. min) and the right dashed line indicating the lambda value for the model where the evaluation metrics are in the range of the best value by one standard error (lambda.1se); **(B)** LASSO regression model screening variable trajectories; **(C)** Boruta; **(D)** common predictors between Boruta and LASSO. LASSO, Least Absolute Shrinkage and Selection Operator.

Feature selection was performed on the training cohort. The LASSO regression algorithm identified 17 key factors, including anemia, hypertension, diabetes, rheumatoid arthritis, kidney disease, liver disease, autoimmune disease, bone metastasis, chemotherapy cycle, chemotherapy regimen, prophylactic medication, and other clinical variables (**Figures 4A, B**). Boruta was utilized to identify characteristic factors such as chemotherapy cycle, chemotherapy regimen, prophylactic medication, ANC, and rescue treatment in the prior cycle (**Figure 4C**). Through a comparative analysis of the outcomes obtained from LASSO regression and Boruta algorithm screening, a common subset of feature variables selected by both methods was identified. These selected features were ultimately utilized in the construction of the model and consisted of chemotherapy cycle, chemotherapy regimen, prophylactic medication, ANC, and rescue treatment in the prior cycle (**Figure 4D**).

The CatBoost model was retrained using the entire training cohort with the selected hyperparameter configuration and subsequently evaluated on the independent testing cohort. The optimized CatBoost model demonstrated good discriminative ability in the independent testing cohort (**Figure 2B**). The receiver operating characteristic (ROC) analysis yielded an AUC of 0.785 (95% CI, 0.706–0.865). The calibration curve was generally close to the reference diagonal, with a Brier score of 0.164, suggesting acceptable overall calibration of the optimized CatBoost model (**Figure 2C**). DCA indicated positive model-based net benefit relative to treat-all and treat-none strategies across the evaluated threshold range (**Figure 2D**). Across a broad range of threshold probabilities, the CatBoost model consistently provided greater net benefit than the treat-all and treat-none strategies, supporting its potential clinical utility for individualized decision-making.

### Model performance under different exploratory operating thresholds

To facilitate different clinical decision-making scenarios, three exploratory probability thresholds were derived from the training cohort, including the Youden index threshold, a screening-oriented threshold, and a confirmatory-oriented threshold. Their predictive performance was subsequently evaluated in the independent testing cohort (**Table 2**). Using the Youden index threshold (0.257), the optimized CatBoost model achieved an accuracy of 0.719 (95% CI, 0.644–0.791), with a sensitivity of 0.750 (95% CI, 0.596,0.891), specificity of 0.706 (95% CI, 0.596–0.808), precision of 0.513 (95% CI, 0.402–0.626), and F1 score of 0.609 (95% CI, 0.496–0.706). At the screening-oriented threshold (0.280), specificity increased to 0.746 (95% CI, 0.645–0.833) while maintaining the same sensitivity, resulting in the highest F1 score and precision. At the confirmatory-oriented threshold (0.377), specificity further increased to 0.794 (95% CI, 0.705–0.872), whereas sensitivity decreased to 0.596 (95% CI, 0.429–0.739), reflecting the expected trade-off between false-positive and false-negative classifications. Confounding matrix is provided in Supplementary Figure I.

**Table 2:** Performance metrics at three thresholds for clinical practice.

Threshold model	Threshold	Accuracy (95% CI)	Sensitivity (95% CI)	Specificity (95% CI)	Precision (95% CI)	F1 (95% CI)
Youden index	0.257	0.719 (0.644,0.791)	0.750 (0.596,0.891)	0.706 (0.596,0.808)	0.513 (0.402,0.626)	0.609 (0.496,0.706)
Screening oriented	0.280	0.747 (0.682,0.811)	0.750 (0.596,0.891)	0.746 (0.645,0.833)	0.549 (0.438,0.654)	0.634 (0.526,0.727)
Confirmatory oriented	0.377	0.736 (0.663,0.810)	0.596 (0.429,0.739)	0.794 (0.705,0.872)	0.544 (0.400,0.672)	0.569 (0.421,0.690)

### Model interpretation analysis

**Figure 5:**
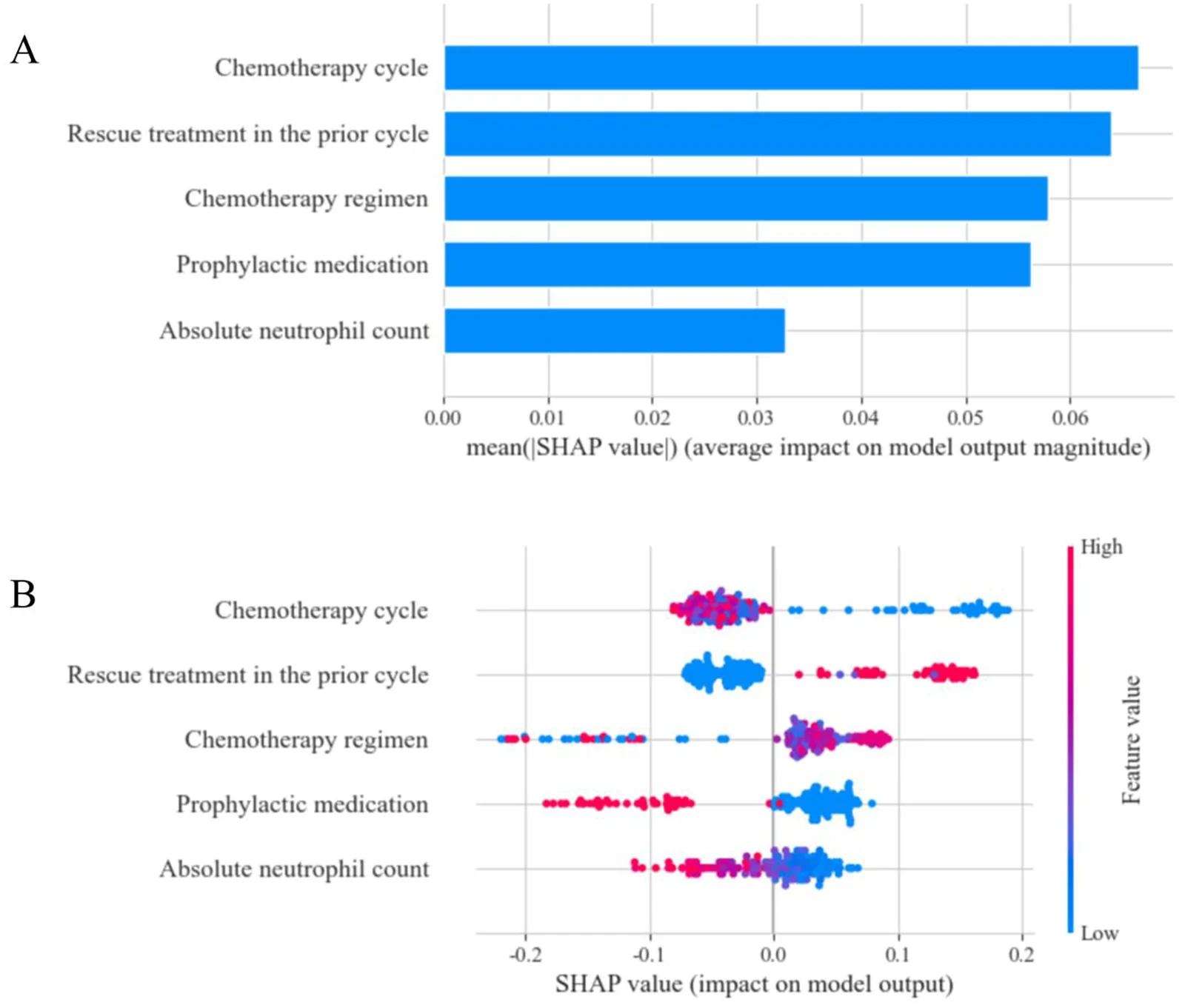
Matrix plots of features. **(A)** a bar plot illustrates the feature importance values determined by SHAP, where a higher value indicates a greater contribution to the model’s predictions. **(B)** a beeswarm plot depicts the distribution of SHAP summary values for each feature across all samples. Each dot represents an individual data point, colored according to its feature value. The x-axis locates the SHAP value, with positive values correlating to features that enhance predictions and negative values associating features that diminish predictions. SHAP, Shapley additive explanations.

**Figure 6:**
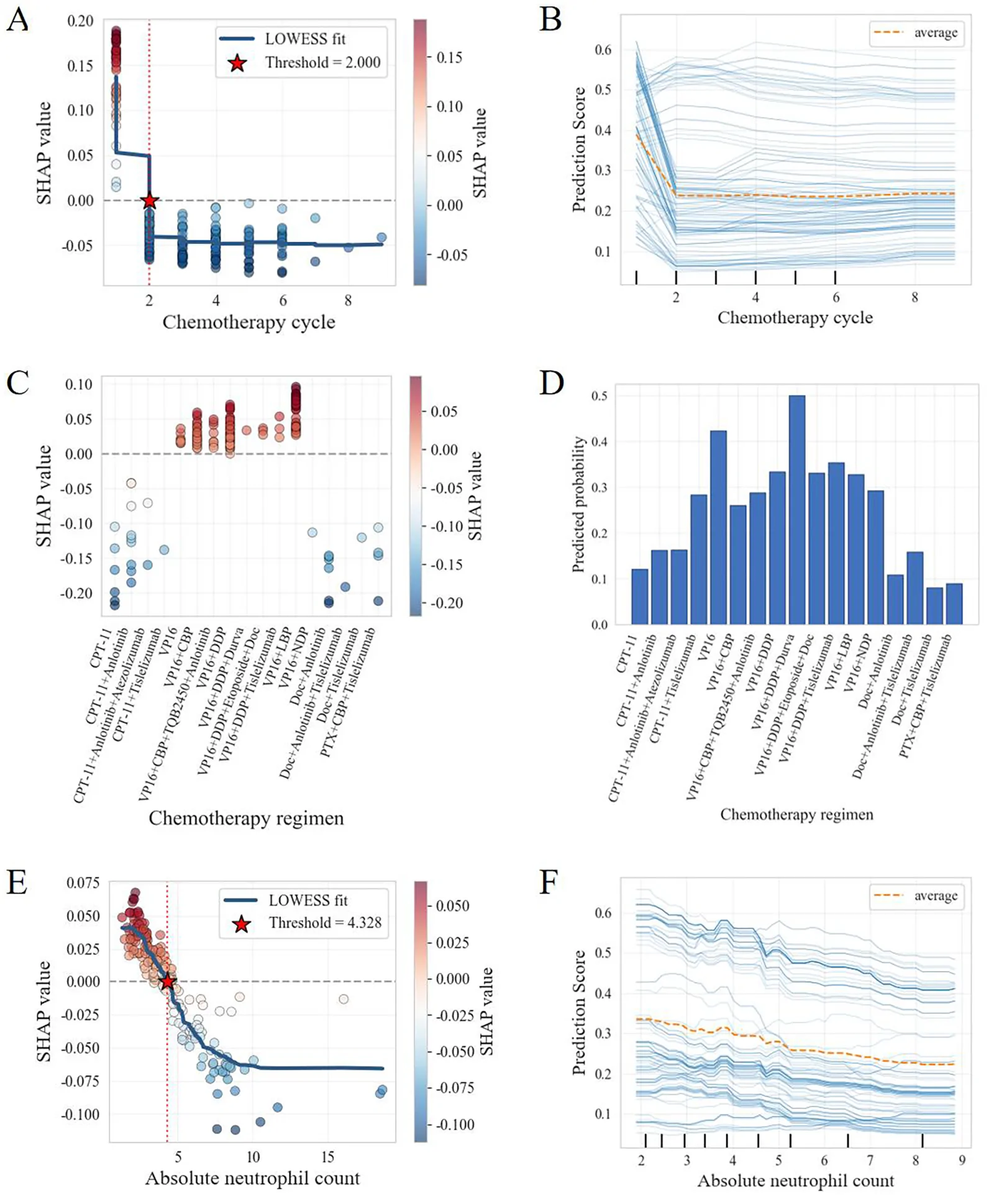
Scatter plot **(A, C, E)** and PDP **(B, D, F)**. **(A, B)** chemotherapy cycle; **(C, D)** chemotherapy regimen; **(E, F)** absolute neutrophil count. CBP, carboplatin; CPT-11, irinotecan; DDP, cisplatin; Doc, docetaxel; LBP, lobaplatin; NDP, nedaplatin; PDP, partial dependence plot; PTX, paclitaxel; SHAP, Shapley additive explanations; VP16, etoposide.

To improve the interpretability of the CatBoost model, SHAP was applied to quantify the contribution of each predictor to the model’s output. The SHAP summary bar plot (**Figure 5A**) ranked the 5 selected features by their mean absolute SHAP values, with chemotherapy cycle showing the strongest overall influence, followed by rescue treatment in the prior cycle, chemotherapy regimen, prophylactic medication, and ANC. The SHAP beeswarm plot (**Figure 5B**) visualized the distribution and direction of each feature’s contribution. Generally, earlier chemotherapy cycle, lower ANC and absence of prophylactic medication were associated with an increased risk of CIN. Rescue treatment in the prior cycle was associated with a higher risk of CIN. Additionally, the VP16 plus platinum regimen was associated with a higher predicted risk of CIN (**Figures 6C, D**).

**Figure 6** presents SHAP-based scatter plots illustrating the associations between key predictors and the CatBoost model output. To facilitate interpretation, PDP and individual conditional expectation (ICE) plots were additionally generated. While ICE plots illustrate prediction variability at the individual level, PDPs summarize the average marginal effect across the study population, providing complementary perspectives on feature-response relationships. The model suggested that the predicted risk declined after the second chemotherapy cycle, indicating that earlier treatment cycles were associated with higher predicted CIN risk (**Figures 6A, B**). Likewise, lower baseline ANC values were associated with higher predicted CIN risk, with a nonlinear relationship observed between baseline ANC and predicted risk (**Figures 6E, F**).

**Figure 7:**
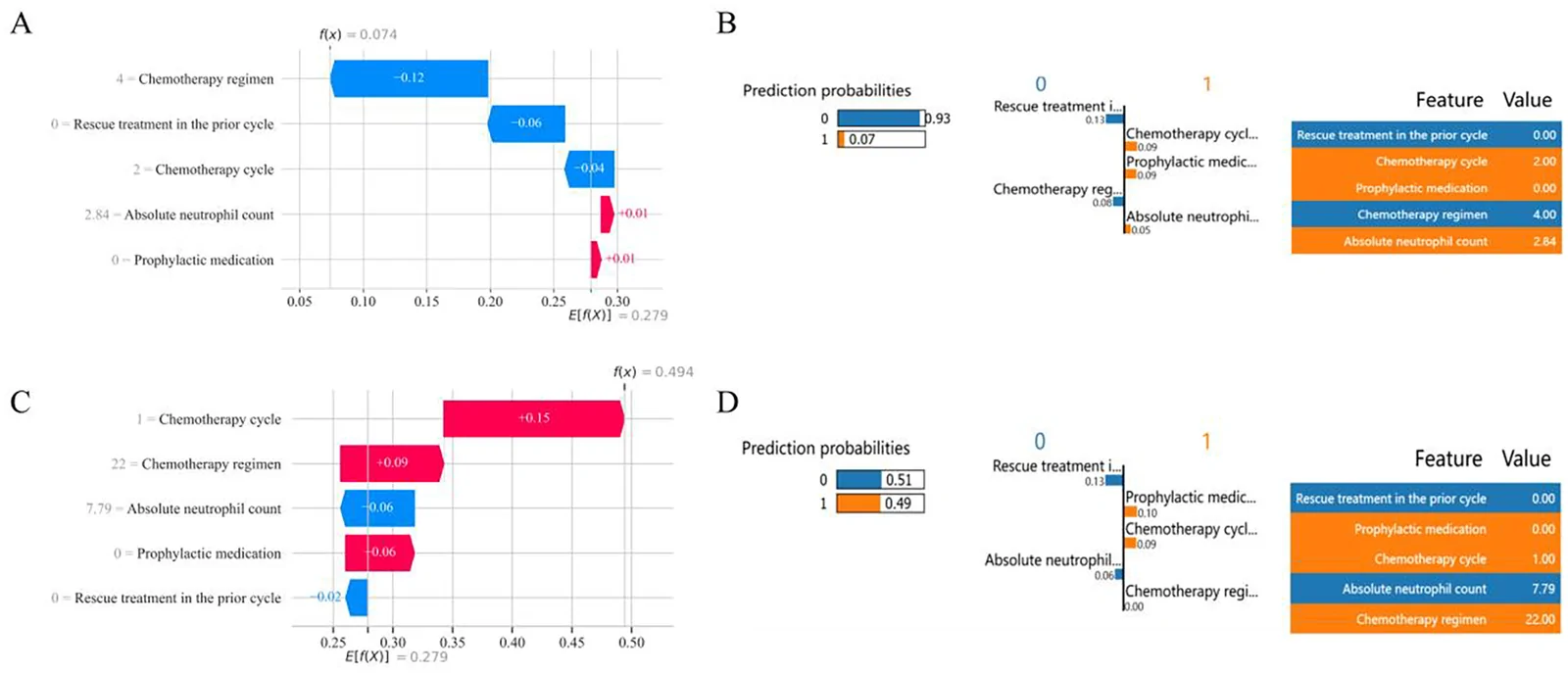
Specific prediction and interpretation of the CatBoost model for two patients. **(A, C)** individual prediction interpretation based on the SHAP method; **(B, D)** individual prediction interpretation based on the LIME method. LIME, Local Interpretable Model-Agnostic Explanations; SHAP, Shapley additive explanations.

Furthermore, the CatBoost model was applied to two representative patients to illustrate individualized risk prediction (**Figure 7**). For the first patient, the predicted probability of developing CIN was 0.07. Both SHAP and LIME explanations indicated that the irinotecan plus anlotinib regimen and the absence of rescue treatment in the prior cycle were the major contributors to the low predicted probability of CIN (**Figures 7A, B**). In contrast, the second patient had a predicted probability of 0.49 for CIN. Earlier chemotherapy cycle and the VP16 plus lobaplatin regimen was identified as the major contributors to the higher predicted probability of CIN (**Figures 7C, D**).

### Web-based clinical decision support tool

**Figure 8:**
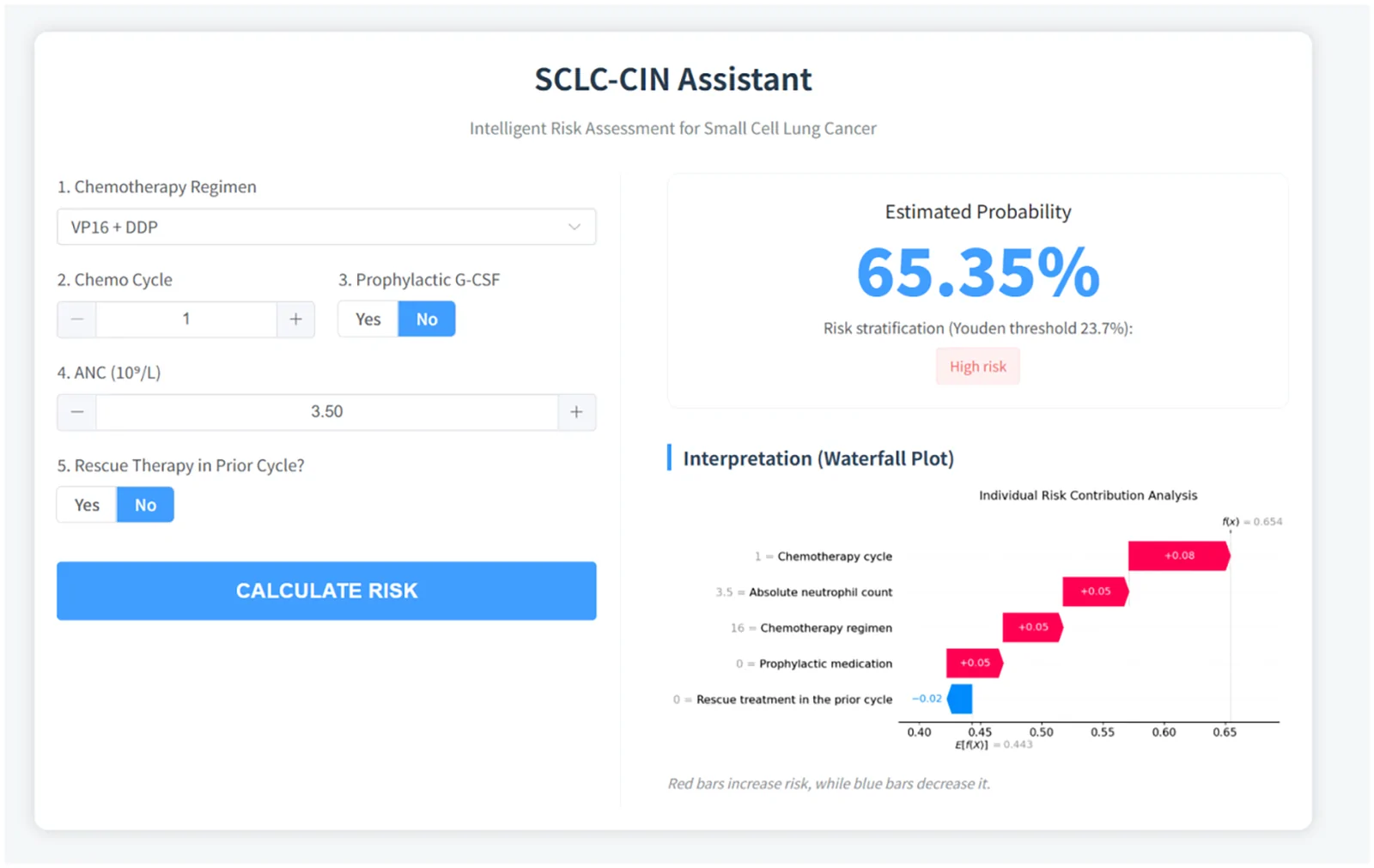
The online clinical decision support tool. Upon setting the required model parameters through the selection panel and clicking ‘Submit’, the application automatically computes and outputs the estimated probability of CIN occurrence based on the user-defined parameters. ANC, absolute neutrophil count.

The CatBoost model has been implemented as a freely accessible web-based application, available at http://39.96.172.15:8080/. As shown in **Figure 8**, users can access the platform via the provided link and interact with the interface by entering the required model parameters. Upon submission, the application generates an individualized risk estimate for CIN. In addition, the system provides a detailed risk report highlighting key risk, along with the relative contribution of each feature to the predicted outcome.

## Discussion

CIN remains a major dose-limiting toxicity of cytotoxic therapy and is particularly relevant in SCLC, where treatment intensity is high and patient vulnerability is substantial (22). Severe neutropenia may compromise treatment delivery through dose reductions or delays and increase the risk of infectious complications (3, 4, 23). In this context, accurate identification of patients at high risk for grade ≥3 CIN may help inform risk stratification and consideration of prophylactic strategies, including G-CSF use and intensified hematologic monitoring. In this study, we developed and validated an interpretable machine learning model for predicting grade ≥3 CIN in patients with SCLC and implemented a web-based clinical decision support tool to facilitate individualized risk assessment. The model demonstrated favorable discriminative performance and provided individualized risk stratification.

Traditional clinical risk scores, such as the FENCE and CSR FENCE models, were developed to predict febrile neutropenia (FN) using conventional regression-based approaches and large population-level datasets (7, 8). These tools incorporate established clinical risk factors, including age, cancer type, treatment regimen, laboratory parameters, comorbidities, and previous chemotherapy exposure, and have demonstrated good applicability for identifying patients at increased risk of FN. However, their reliance on additive linear effects limits their ability to capture complex nonlinear interactions among predictors, and their primary endpoint of FN may not fully reflect the broader spectrum of clinically significant chemotherapy-induced neutropenia. Because severe CIN frequently precedes FN and often prompts chemotherapy dose modification or prophylactic intervention before infection develops, predicting grade ≥3 CIN may provide an earlier opportunity for individualized supportive care. More recently, machine learning has been increasingly applied to improve neutropenia risk prediction. Wiberg et al. developed a machine learning framework for predicting neutropenic events using routinely collected electronic health record data and demonstrated that ensemble learning algorithms outperformed conventional statistical models by exploiting complex relationships among multiple clinical variables (9). Similarly, Venäläinen et al. constructed and externally validated a real-world machine learning model for CIN prediction, showing improved discrimination compared with traditional approaches and highlighting the value of large-scale clinical data for individualized risk assessment (10). These studies collectively demonstrate the promise of machine learning in enhancing predictive performance. However, most of these models were developed in heterogeneous cancer populations, included multiple chemotherapy regimens across different malignancies, and provided limited model interpretability or disease-specific clinical implementation. Our study addresses these gaps by focusing on a homogeneous SCLC population, using a well-defined chemotherapy backbone, and integrating comprehensive interpretability analyses with a deployable clinical decision support tool.

The model behavior was directionally consistent with some previously reported clinical patterns. Notably, the chemotherapy cycle emerged as the most significant predictor, with SHAP analysis revealing a pronounced decrease in risk after the second cycle. Previous studies have shown that the risk of CIN is highest during the initial cycles of treatment, prior to dose modification or implementation of supportive care measures (8, 24). This observation suggests that early treatment exposure may reveal interindividual susceptibility to myelosuppression. These findings may help inform risk stratification during the early treatment phase, although whether such risk stratification can improve prophylactic management requires prospective evaluation. The association between prior-cycle rescue treatment and increased CIN risk highlights the dynamic nature of toxicity susceptibility. Previous studies suggest that prior hematologic toxicity is a strong predictor of subsequent adverse events, reflecting cumulative or intrinsic vulnerability (8, 24). The inverse association between prophylactic medication and CIN risk observed in our study is consistent with guideline recommendations supporting the use of G-CSF in high-risk patients to reduce the incidence of severe neutropenia (25). Furthermore, VP16 plus platinum-based chemotherapy was associated with increased CIN risk, consistent with its well-documented myelosuppressive profile. A clinical study in Japanese patients with small-cell lung cancer demonstrated that VP16 plus platinum regimens were associated with a 62% incidence of grade 3–4 neutropenia (26).

Baseline hematologic reserve, reflected by the ANC, showed an inverse association with the predicted risk of CIN. This finding is consistent with previous studies reporting. In a cohort of 186 breast cancer patients not receiving primary G-CSF prophylaxis, pre-treatment ANC was significantly associated with grade 4 CIN risk in both the first cycle (OR = 0.68, p=0.006) and any cycle (OR = 0.78, p=0.032) (27). The biological plausibility of this association may be related to the relationship between circulating neutrophil levels and the size of the bone marrow granulocyte reserve pool. Patients with relatively low baseline ANC may have a smaller pre-existing bone marrow reserve, which could make them more susceptible to myelosuppressive chemotherapy. This interpretation is supported by early studies demonstrating that patients with normal baseline leukocyte counts can still have impaired bone marrow granulocyte reserve as measured by prednisolone stimulation tests (28). Further studies are needed to clarify the biological and clinical significance of baseline ANC in predicting CIN.

Compared with traditional statistical approaches, our study leveraged machine learning techniques to capture complex, nonlinear relationships among clinical and laboratory variables. A dual feature selection strategy combining LASSO regression and the Boruta algorithm was used to combine complementary linear and nonlinear feature-selection criteria (14, 29). Among the evaluated models, CatBoost was selected as the optimal algorithm. The optimized CatBoost model achieved an AUC of 0.785 (95% CI, 0.706–0.865. This favorable outcome confirms the efficacy of ensemble machine learning for clinical prediction. Compared with conventional regression-based models, the CatBoost model demonstrated robust predictive performance while reducing feature redundancy and enhancing model stability (30). Additionally, we explored three probability thresholds as exploratory operating points: the Youden-index threshold, a screening-oriented threshold targeting sensitivity ≥0.75, and a confirmatory-oriented threshold targeting specificity ≥0.80. These pragmatic targets were prespecified to represent relatively high sensitivity for screening and relatively high specificity for confirmatory assessment, rather than being derived from established clinical guidelines or representing clinically established decision thresholds. In the independent testing cohort, the screening-oriented threshold preserved the same sensitivity as the Youden-index threshold while improving specificity. By contrast, the confirmatory-oriented threshold further reduced false-positive predictions at the expense of lower sensitivity, illustrating the expected trade-off between sensitivity and specificity. These findings demonstrate the potential flexibility of threshold selection according to different operating objectives, but the proposed thresholds should be considered exploratory and require prospective validation before clinical implementation.

A key strength of this study lies in its emphasis on model interpretability. Many machine learning models function as ‘black boxes’, thereby limiting their clinical applicability (31, 32). To address this limitation, we incorporated SHAP, LIME, and PDP to provide both global and individualized explanations of model behavior. These methods enabled identification of key predictors and clarification of their directional effects, thereby enhancing model transparency and facilitating the interpretation of individual predictions. Another important contribution is the development of a web-based clinical decision support system. Such tools can facilitate real-time risk prediction at the point of care and may help translate predictive models into potential clinical applications (33). Despite the growing number of predictive models in oncology, relatively few have been implemented as point-of-care tools. Our platform enables clinicians to input patient-specific variables and obtain real-time risk predictions with interpretable outputs. This integration of prediction, explanation, and usability represents a step toward potential real-world clinical application. However, the clinical utility and impact of the tool remain to be established through external validation and prospective evaluation.

Several limitations should be acknowledged. First, this was a single-center study, which may limit generalizability across different populations and treatment settings. Second, although internal validation was rigorously performed using cross-validation, external validation in independent multicenter cohorts is necessary to confirm model transportability. Third, the model was based on routinely available clinical variables; incorporation of additional data modalities, such as genomic, inflammatory, or pharmacokinetic markers, may further improve predictive performance. Finally, although the interpretability framework provides valuable insights into model behavior, the causal relationships underlying these associations require prospective validation.

In conclusion, we developed and validated an interpretable machine learning model for predicting grade ≥3 CIN in patients with SCLC. By identifying key predictors—including early chemotherapy cycles, baseline ANC, and prophylactic medication use—this study provides information that may support individualized risk stratification. The integration of the model into a web-based clinical decision support tool enables individualized risk estimation with interpretable outputs and represents a potential step toward clinical application. However, the potential utility of the model for informing prophylactic interventions, including G-CSF use, or improving treatment continuity and clinical outcomes remains to be established. External validation in independent, multicenter cohorts and prospective evaluation of clinical impact are warranted before broader clinical implementation.

## Data Availability

The raw data supporting the conclusions of this article will be made available by the authors, without undue reservation.
