# Supplementary material for: Development and validation of an interpretable machine learning model for predicting chemotherapy-induced neutropenia in small cell lung cancer: a web-based clinical decision support tool
Source: Front Oncol. 2026 Sep 4;16:1939436. doi: 10.3389/fonc.2026.1939436 (PMC13585533; doi:10.3389/fonc.2026.1939436)
Supplement: Supplementary file 1 [file DataSheet1.docx]

**Supplemental Material**

**Development and validation of an interpretable machine learning model for predicting chemotherapy-induced neutropenia in small cell lung cancer: a web-based clinical decision support tool**

**Supplemental Tables**

I. **Missing values**
II. **Digital conversion of categorial variables**

**III. Details concerning the formulas for each evaluation metric**

**Supplemental Figures**

**I. Confounding matrix of the CatBoost model**

**Supplemental Tables**

Table I. Missing values

| **Variables** | **Variable type** | **Missing values N (%)** |
| --- | --- | --- |
| Body surface area | Continuous | 5(0.6**%**) |
| Aspartate aminotransferase | Continuous | 95(11.3**%**) |
| Alanine aminotransferase | Continuous | 95(11.3**%**) |
| Alkaline phosphatase | Continuous | 103(12.3**%**) |
| Gamma-glutamyl transferase | Continuous | 102(12.1**%**) |
| Plasma bilirubin | Continuous | 101(12.0**%**) |
| Total Protein | Continuous | 99(11.8**%**) |
| Albumin | Continuous | 94(11.2**%**) |
| Serum creatinine | Continuous | 97(11.5**%**) |
| Glomerular filtration rate | Continuous | 104(12.4**%**) |
| Leukocyte count | Continuous | 11(1.3**%**) |
| Absolute neutrophil count | Continuous | 11(1.3**%**) |
| Lymphocyte count | Continuous | 15(1.8**%**) |
| Blood hemoglobin | Continuous | 11(1.3**%**) |
| Thrombocyte count | Continuous | 12(1.4**%**) |
| CIN in the prior cycle | Categorial | 21(2.5%) |
| Rescue treatment in the prior cycle | Categorial | 21(2.5%) |

.

Table II. Digital conversion of categorial variables

| **Categorial variables** | **Digital conversion** |
| --- | --- |
| Gender | 0-Female,1-Male |
| Chemotherapy regimen | 0-CPT-11, 1-CPT-11+CBP, 2-CPT-11+DDP, 3-CPT-11+LBP, 4-CPT-11+Anlotinib, 5-CPT-11+Anlotinib+Atezolizumab, 6-CPT-11+Tislelizumab, 7-CPT-11+Apatinib, 8-CPT-11+Atezolizumab, 9-VP16, 10-VP16+CBP, 11-VP16+CBP+Durva, 12-VP16+CBP+Gef, 13-VP16+CBP+TQB2450+Anlotinib, 14-VP16+CBP+Crizotinib, 15-VP16+CBP+Atezolizumab, 16-VP16+DDP 17-VP16+DDP+Durva, 18-VP16+DDP+Etoposide, 19-VP16+DDP+Etoposide+Doc, 20-VP16+DDP+Tislelizumab, 21-VP16+DDP+Atezolizumab, 22-VP16+LBP, 23-VP16+LBP+Atezolizumab, 24-VP16+NDP, 25-VP16+Tislelizumab, 26-Doc, 27-Doc+Anlotinib, 28-Doc+Anlotinib+Tislelizumab, 29-Doc+Tislelizumab, 30-Doc+Atezolizumab, 31-PTX, 32-PTX+CBP+Tislelizumab |
| Anaemia | 0-No, 1-Yes |
| Hypertension | 0-No, 1-Yes |
| Lung disease | 0-No, 1-Yes |
| Diabetes | 0-No, 1-Yes |
| Thyroid disease | 0-No, 1-Yes |
| Heart failure | 0-No, 1-Yes |
| Rheumatoid arthritis | 0-No, 1-Yes |
| Kidney disease | 0-No, 1-Yes |
| Liver disease | 0-No, 1-Yes |
| Autoimmune disease | 0-No, 1-Yes |
| Coronary heart disease | 0-No, 1-Yes |
| Bone metastasis | 0-No, 1-Yes |
| Prophylactic medication | 0-No, 1-Yes |
| CIN in the prior cycle | 0-No, 1-Yes |
| Rescue treatment in the prior cycle | 0-No, 1-Yes |

Abbreviations; CBP, carboplatin; DDP, cisplatin; LBP, lobaplatin; NDP, nedaplatin; Doc, docetaxel; PTX, paclitaxel; VP16, etoposide; CPT11, irinotecan.

Table III Details concerning the formulas for each evaluation metric

| **Index** | **Formula** |
| --- | --- |
| Accuracy | (TP+TN)/(TP+TN+FP+FN) |
| Sensitivity | TP/(TP+FN) |
| Precision | TP/(TP+FP) |
| F1 score | 2/F1=1/Precision+1/Sensitivity |
| Specificity | TN/(TN+FP) |

Abbreviations; FN, false negatives; FP, false positives; TN, true negatives; TP, true positives.

Supplementary Figure I. Confounding matrix of the CatBoost model. (A) Confounding matrix of the Youden index threshold model, (B) Confounding matrix of the screening oriented threshold model, (C) Confounding matrix of the confirmatory oriented threshold model. Abbreviations; CatBoost, categorical boosting.

.
